# Supplementary material for: Partial Disturbance of Microprocessor Function in Human Stem Cells Carrying a Heterozygous Mutation in the DGCR8 Gene
Source: Genes (Basel). 2022 Oct 23;13(11):1925. doi: 10.3390/genes13111925 (PMC9689658; doi:10.3390/genes13111925)
Supplement: Supplementary file 1 [file genes-13-01925-s001.zip › Figure S1 Ree et al.pdf]

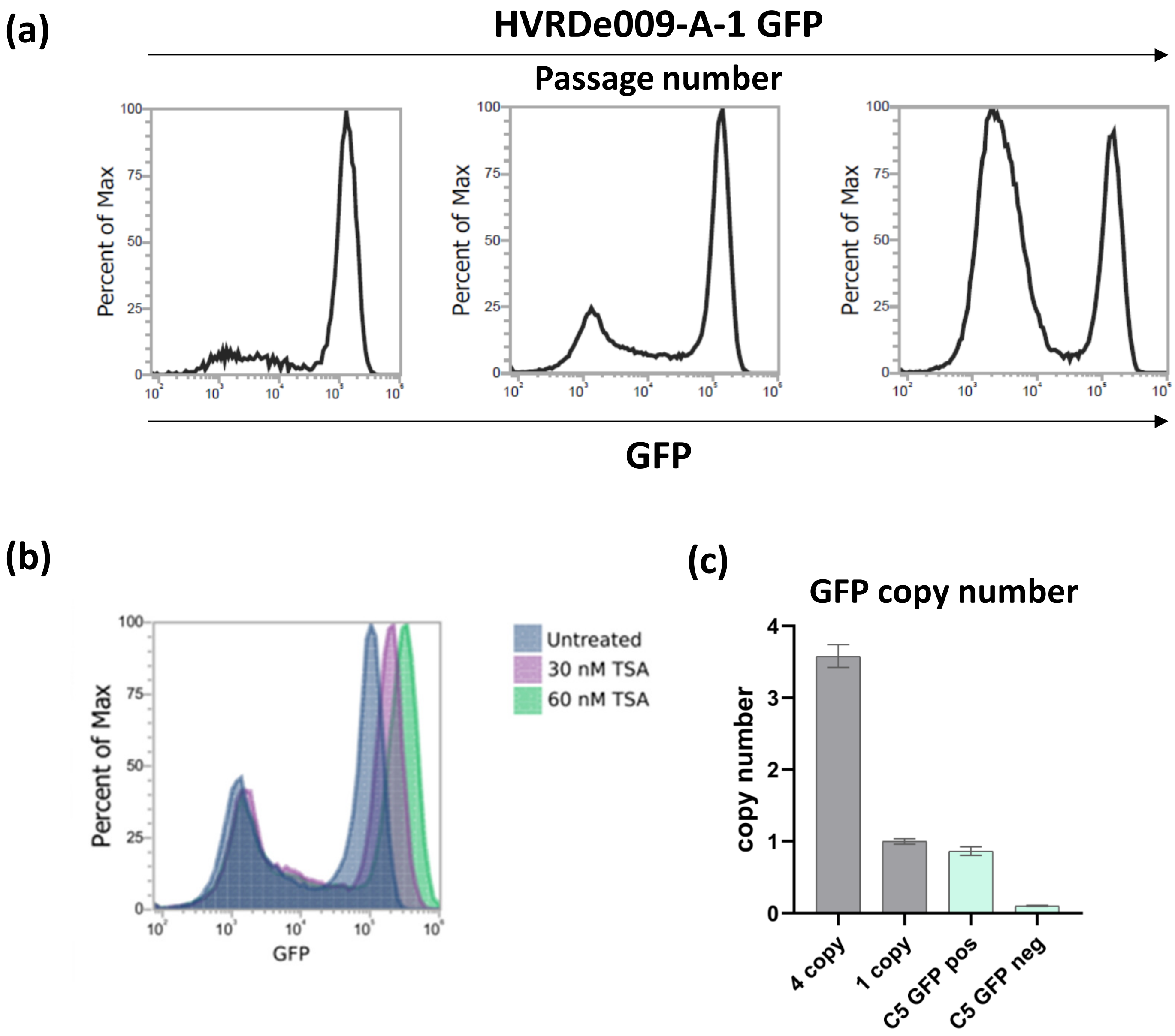

Supplementary Figure S1. GFP expression in the HVRDe009-A-1 cell line. (a) GFP FACS measurements of the cells during puromycin deprivation. **(b)** GFP FACS measurements of the cells treated with 0, 30 or 60 nM Trichostatin A (TSA), respectively. **(c)** GFP copy number measurements in the GFP positive or negative sorted populations. Relative quantitation (RQ) values were calculated using RPPH1 as reference target and a 1 copy control as reference sample.
