# Supplementary material for: Partial Disturbance of Microprocessor Function in Human Stem Cells Carrying a Heterozygous Mutation in the DGCR8 Gene
Source: Genes (Basel). 2022 Oct 23;13(11):1925. doi: 10.3390/genes13111925 (PMC9689658; doi:10.3390/genes13111925)
Supplement: Supplementary file 1 [file genes-13-01925-s001.zip › Figure S2 Ree et al.pdf]

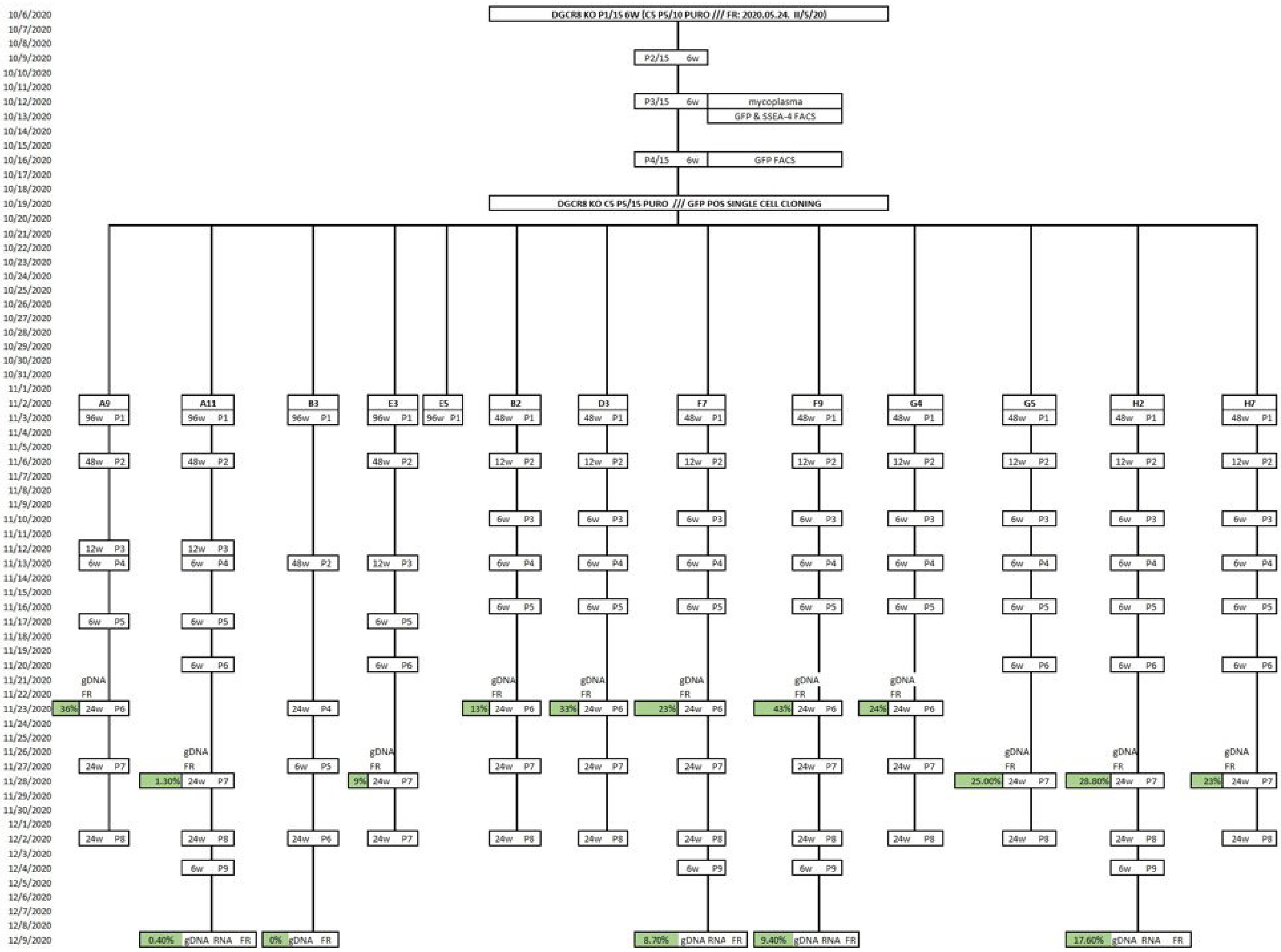

Supplementary Figure S2. Overview of the establishment of "GFP positive" single cell clones from the HVRDe009-A-1 cell line during puromycin deprivation. GFP FACS measurement time points labelled green with the corresponding percentage of the GFP expressing cells in the culture.
