# Supplementary material for: Partial Disturbance of Microprocessor Function in Human Stem Cells Carrying a Heterozygous Mutation in the DGCR8 Gene
Source: Genes (Basel). 2022 Oct 23;13(11):1925. doi: 10.3390/genes13111925 (PMC9689658; doi:10.3390/genes13111925)
Supplement: Supplementary file 1 [file genes-13-01925-s001.zip › Figure S4 Ree et al_revised .pdf]

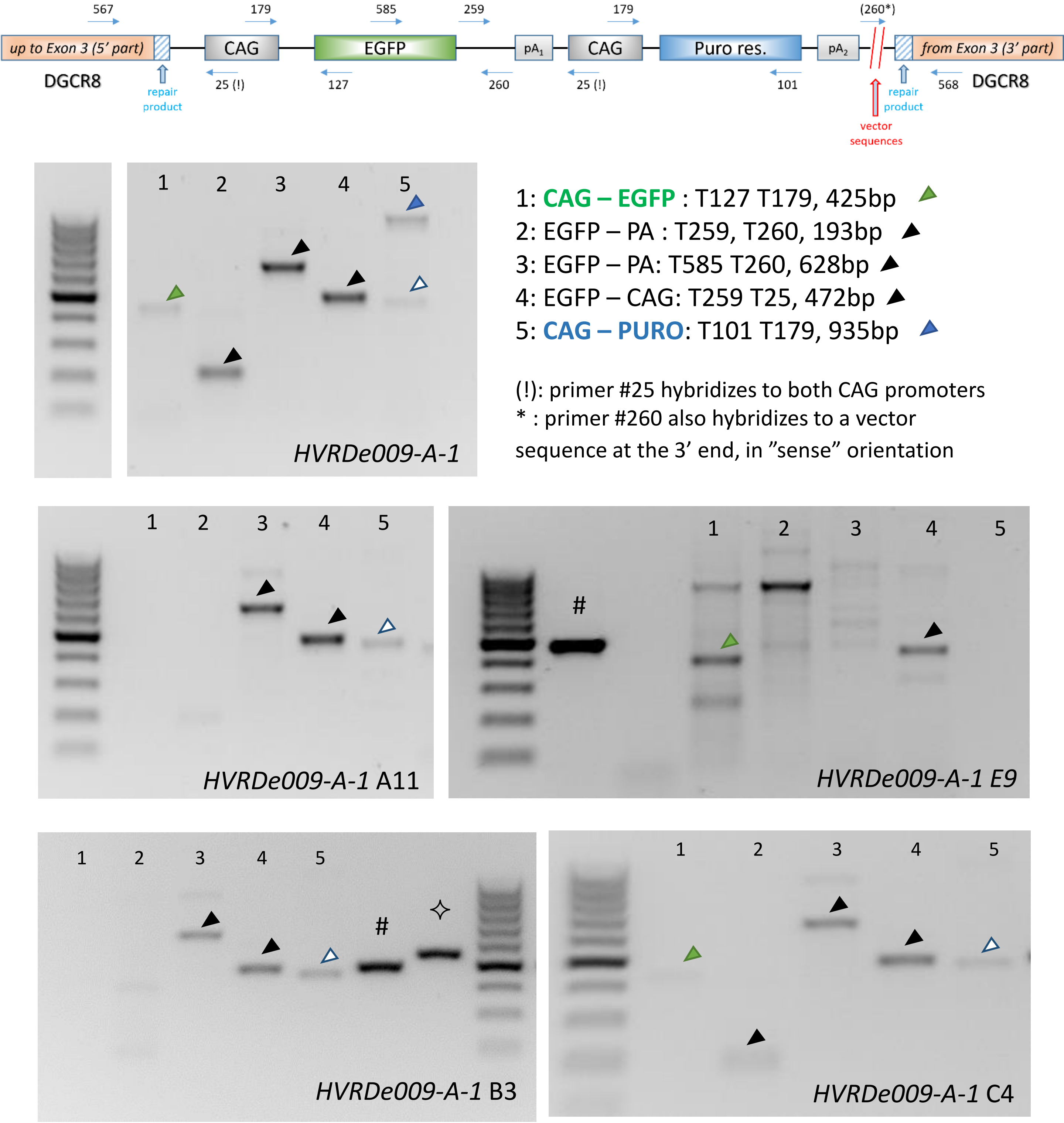

Supplementary Figure S4. Diagnostic PCR results in the parental HVRDe009-A-1 cell line and the derived single cell clones. Each primer set covers a different fragment in the inserted cassette. Green black and blue arrows indicate the expected amplicon sizes. #: amplifying the normal allele (with primers 567+568);

✧ : amplifying the 5' junction of the CRISPR-modified allele (with primers 567+25).
