# Supplementary material for: Partial Disturbance of Microprocessor Function in Human Stem Cells Carrying a Heterozygous Mutation in the DGCR8 Gene
Source: Genes (Basel). 2022 Oct 23;13(11):1925. doi: 10.3390/genes13111925 (PMC9689658; doi:10.3390/genes13111925)
Supplement: Supplementary file 1 [file genes-13-01925-s001.zip › Figure S5 Ree et al_revised.pdf]

(a) 5' sequence of the integrated transgene - a schematic overview

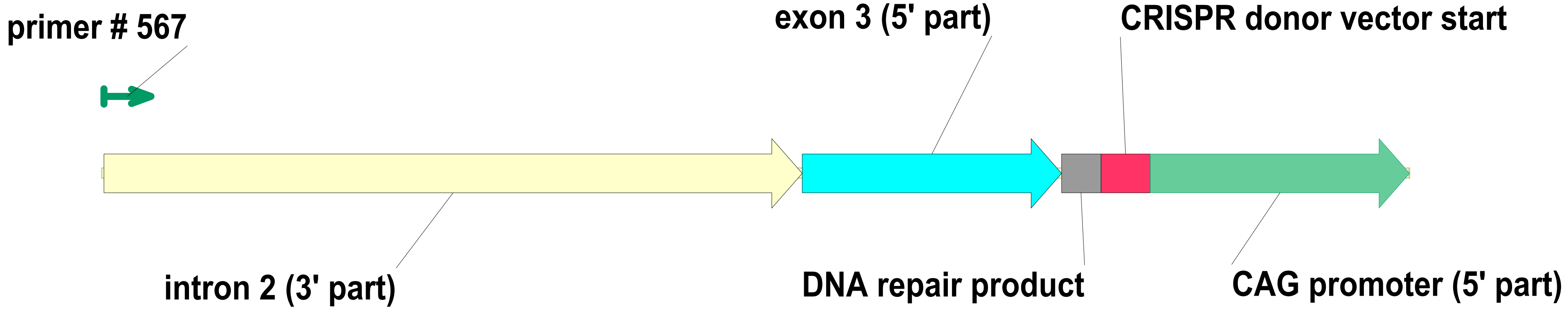

(b)

DGCR8 mutant allele sequenced in the clones (page 1)

```
                                                                          Section 1
                                   (1)   1       10        20        30       40
                     A11 clone     (1)   ----------------------------------------
                      B3 clone     (1)   ----------------------------------------
                      C4 clone     (1)   ----------------------------------------
                      E9 clone     (1)   ----------------------------------------
C5 clone transgene integration 5' end (1)   AGTTTGGCCCATGGGTAGGCCCTGCATCCCTGATCTAGCG
                     Consensus     (1)
                                                                          Section 2
                                  (41)   41      50        60        70       80
                     A11 clone     (1)   ---------GCAGGTGCTGCTGAGTTACGCTCCTTGGCAG
                      B3 clone     (1)   ---------GCAGGTGCTGCTGAGTTACGCTCCTTGGCAG
                      C4 clone     (1)   ---------GCAGGTGCTGCTGAGTTACGCTCCTTGGCAG
                      E9 clone     (1)   ---------GCAGGTGCTGCTGAGTTACGCTCCTTGGCAG
C5 clone transgene integration 5' end (41)  CGTGGGGCAGCAGGTGCTGCTGAGTTACGCTCCTTGGCAG
                     Consensus    (41)            GCAGGTGCTGCTGAGTTACGCTCCTTGGCAG
                                                                          Section 3
                                  (81)   81      90        100       110      120
                     A11 clone    (32)   TGTGTGCCCCTGGACCAGGTGTGTTGGTGTCAGCTGGTAG
                      B3 clone    (32)   TGTGTGCCCCTGGACCAGGTGTGTTGGTGTCAGCTGGTAG
                      C4 clone    (32)   TGTGTGCCCCTGGACCAGGTGTGTTGGTGTCAGCTGGTAG
                      E9 clone    (32)   TGTGTGCCCCTGGACCAGGTGTGTTGGTGTCAGCTGGTAG
C5 clone transgene integration 5' end (81)  TGTGTGCCCCTGGACCAGGTGTGTTGGTGTCAGCTGGTAG
                     Consensus    (81)   TGTGTGCCCCTGGACCAGGTGTGTTGGTGTCAGCTGGTAG
                                                                          Section 4
                                 (121)   121     130       140       150      160
                     A11 clone    (72)   CTTCATCCTGTTTGTTTTTCAGATGATCATGCACCCTAAG
                      B3 clone    (72)   CTTCATCCTGTTTGTTTTTCAGATGATCATGCACCCTAAG
                      C4 clone    (72)   CTTCATCCTGTTTGTTTTTCAGATGATCATGCACCCTAAG
                      E9 clone    (72)   CTTCATCCTGTTTGTTTTTCAGATGATCATGCACCCTAAG
C5 clone transgene integration 5' end (121) CTTCATCCTGTTTGTTTTTCAGATGATCATGCACCCTAAG
                     Consensus   (121)   CTTCATCCTGTTTGTTTTTCAGATGATCATGCACCCTAAG
                                                                          Section 5
                                 (161)   161     170       180       190      200
                     A11 clone   (112)   GGCACATCTAGGCCCCCTGAGAGCACCTCCTTTCTGTGTC
                      B3 clone   (112)   GGCACATCTAGGCCCCCTGAGAGCACCTCCTTTCTGTGTC
                      C4 clone   (112)   GGCACATCTAGGCCCCCTGAGAGCACCTCCTTTCTGTGTC
                      E9 clone   (112)   GGCACATCTAGGCCCCCTGAGAGCACCTCCTTTCTGTGTC
C5 clone transgene integration 5' end (161) GGCACATCTAGGCCCCCTGAGAGCACCTCCTTTCTGTGTC
                     Consensus   (161)   GGCACATCTAGGCCCCCTGAGAGCACCTCCTTTCTGTGTC
                                                                          Section 6
                                 (201)   201     210       220       230      240
                     A11 clone   (152)   TGTTCTCAGGAATGCTGTTGAGCTCTCCTGTTGCAGGAGC
                      B3 clone   (152)   TGTTCTCAGGAATGCTGTTGAGCTCTCCTGTTGCAGGAGC
                      C4 clone   (152)   TGTTCTCAGGAATGCTGTTGAGCTCTCCTGTTGCAGGAGC
                      E9 clone   (152)   TGTTCTCAGGAATGCTGTTGAGCTCTCCTGTTGCAGGAGC
C5 clone transgene integration 5' end (201) TGTTCTCAGGAATGCTGTTGAGCTCTCCTGTTGCAGGAGC
                     Consensus   (201)   TGTTCTCAGGAATGCTGTTGAGCTCTCCTGTTGCAGGAGC
```

DGCR8 mutant allele sequenced in the clones (page 2)

Section 7

(241)	241 250 260 270 280
A11 clone (192)	ATGAGCGCCAGGGGCTCTGGTGTCTGAACAGCGTGTTTTG
B3 clone (192)	ATGAGCGCCAGGGGCTCTGGTGTCTGAACAGCGTGTTTTG
C4 clone (192)	ATGAGCGCCAGGGGCTCTGGTGTCTGAACAGCGTGTTTTG
E9 clone (192)	ATGAGCGCCAGGGGCTCTGGTGTCTGAACAGCGTGTTTTG
C5 clone transgene integration 5' end (241)	ATGAGCGCCAGGGGCTCTGGTGTCTGAACAGCGTGTTTTG
Consensus (241)	ATGAGCGCCAGGGGCTCTGGTGTCTGAACAGCGTGTTTTG

Section 8

(281)	281 290 300 310 320
A11 clone (232)	CAGGATGACTTTGACAACGATGTGGATGCTCTGCTGGAAG
B3 clone (232)	CAGGATGACTTTGACAACGATGTGGATGCTCTGCTGGAAG
C4 clone (232)	CAGGATGACTTTGACAACGATGTGGATGCTCTGCTGGAAG
E9 clone (232)	CAGGATGACTTTGACAACGATGTGGATGCTCTGCTGGAAG
C5 clone transgene integration 5' end (281)	CAGGATGACTTTGACAACGATGTGGATGCTCTGCTGGAAG
Consensus (281)	CAGGATGACTTTGACAACGATGTGGATGCTCTGCTGGAAG

Section 9

(321)	321 330 340 350 360
A11 clone (272)	AAGGCCTTTGTGCCCCCAAAAAGAGGCGAACAGAGGAAAA
B3 clone (272)	AAGGCCTTTGTGCCCCCAAAAAGAGGCGAACAGAGGAAAA
C4 clone (272)	AAGGCCTTTGTGCCCCCAAAAAGAGGCGAACAGAGGAAAA
E9 clone (272)	AAGGCCTTTGTGCCCCCAAAAAGAGGCGAACAGAGGAAAA
C5 clone transgene integration 5' end (321)	AAGGCCTTTGTGCCCCCAAAAAGAGGCGAACAGAGGAAAA
Consensus (321)	AAGGCCTTTGTGCCCCCAAAAAGAGGCGAACAGAGGAAAA

Section 10

(361)	361 370 380 390 400
A11 clone (312)	ATATGGCGGAGACAGCGACCATCCGTCCTAATCGAATTCA
B3 clone (312)	ATATGGCGGAGACAGCGACCATCCGTCCTAATCGAATTCA
C4 clone (312)	ATATGGCGGAGACAGCGACCATCCGTCCTAATCGAATTCA
E9 clone (312)	ATATGGCGGAGACAGCGACCATCCGTCCTAATCGAATTCA
C5 clone transgene integration 5' end (361)	ATATGGCGGAGACAGCGACCATCCGTCCTAATCGAATTCA
Consensus (361)	ATATGGCGGAGACAGCGACCATCCGTCCTAATCGAATTCA

Section 11

(401)	401 410 420 430 440
A11 clone (352)	CTAGTCGAATTCACTAGTGCGCGCGGCCGCTCTAGCCCCT
B3 clone (352)	CTAGTCGAATTCACTAGTGCGCGCGGCCGCTCTAGCCCCT
C4 clone (352)	CTAGTCGAATTCACTAGTGCGCGCGGCCGCTCTAGCCCCT
E9 clone (352)	CTAGTCGAATTCACTAGTGCGCGCGGCCGCTCTAGCCCCT
C5 clone transgene integration 5' end (401)	CTAGTCGAATTCACTAGTGCGCGCGGCCGCTCTAGCCCCT
Consensus (401)	CTAGTCGAATTCACTAGTGCGCGCGGCCGCTCTAGCCCCT

Section 12

(441)	441 450 460 470 480
A11 clone (392)	AGTTATTAATAGTAATCAATTACGGGGTCATTAGTTCATA
B3 clone (392)	AGTTATTAATAGTAATCAATTACGGGGTCATTAGTTCATA
C4 clone (392)	AGTTATTAATAGTAATCAATTACGGGGTCATTAGTTCATA
E9 clone (392)	AGTTATTAATAGTAATCAATTACGGGGTCATTAGTTCATA
C5 clone transgene integration 5' end (441)	AGTTATTAATAGTAATCAATTACGGGGTCATTAGTTCATA
Consensus (441)	AGTTATTAATAGTAATCAATTACGGGGTCATTAGTTCATA

Supplementary Figure S5. Sanger sequencing of the DGCR8 mutant allele in the HVRDe009-A-1 derived single cell clones. (**a**) Schematic representation of the 5' transgene sequence environment. The indicated primer in intron 2 was used for sequencing; the different regions are marked with different colors. (**b**) Sequence alignment of the 5′ integration site of the transgene in the examined clones. The 5′ residual part of DGCR8 exon 3 sequence is labeled with light blue; the DNA repair product at the Cas9 cleavage site is labeled with grey; the start of the CRISPR donor vector sequence is labeled with red; the start of the first CAG promoter is labeled with green.
