# Supplementary material for: Partial Disturbance of Microprocessor Function in Human Stem Cells Carrying a Heterozygous Mutation in the DGCR8 Gene
Source: Genes (Basel). 2022 Oct 23;13(11):1925. doi: 10.3390/genes13111925 (PMC9689658; doi:10.3390/genes13111925)
Supplement: Supplementary file 1 [file genes-13-01925-s001.zip › Figure S6 Ree et al.pdf]

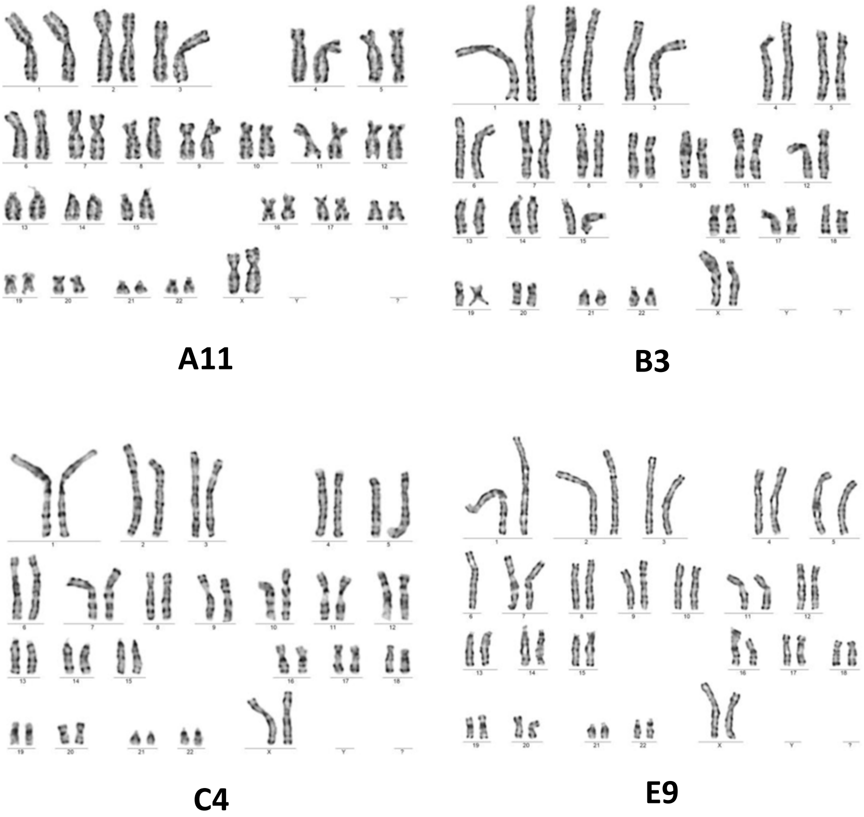

Supplementary Figure S6. Representative karyograms of the HVRDe009-A-1 derived single cell clones. Resolution 450–500 bands per haploid chromosome set.
