# Supplementary material for: Partial Disturbance of Microprocessor Function in Human Stem Cells Carrying a Heterozygous Mutation in the DGCR8 Gene
Source: Genes (Basel). 2022 Oct 23;13(11):1925. doi: 10.3390/genes13111925 (PMC9689658; doi:10.3390/genes13111925)
Supplement: Supplementary file 1 [file genes-13-01925-s001.zip › Figure S7 Ree et al_revised.pdf]

(a)

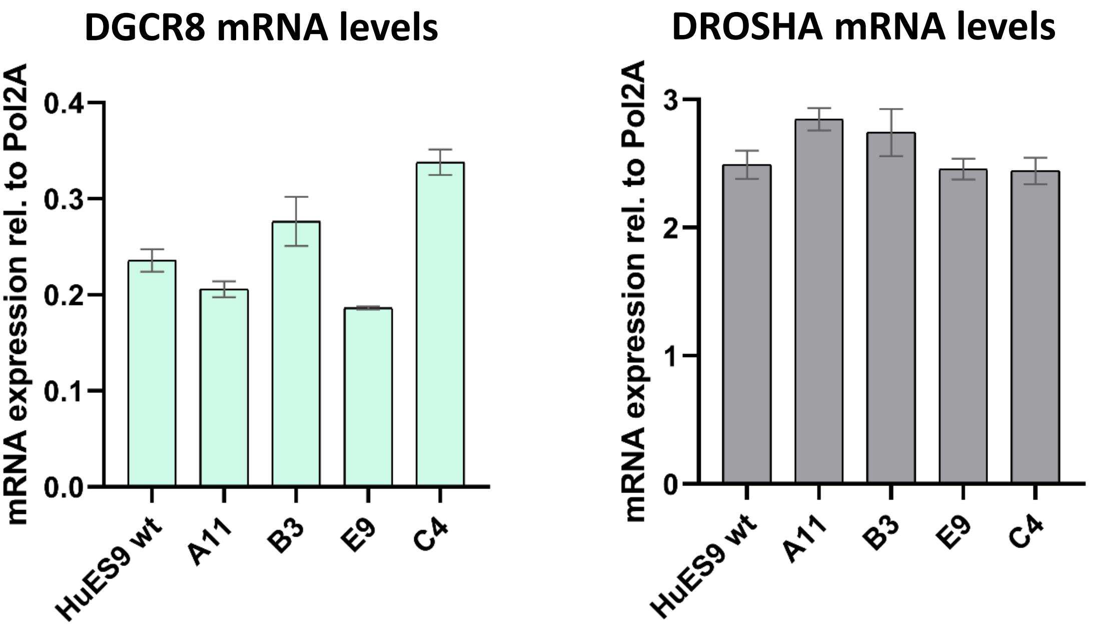

Other representative Western blots

(b)

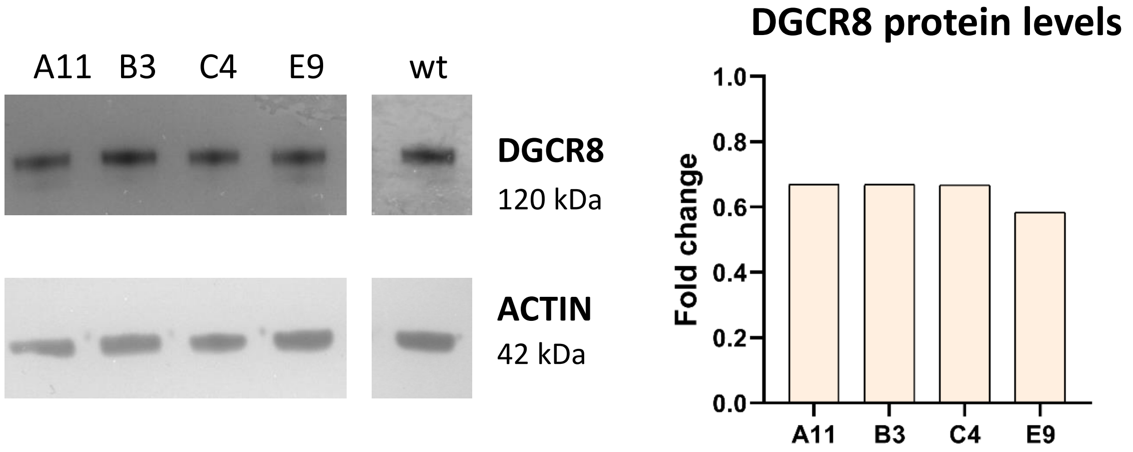

(c)

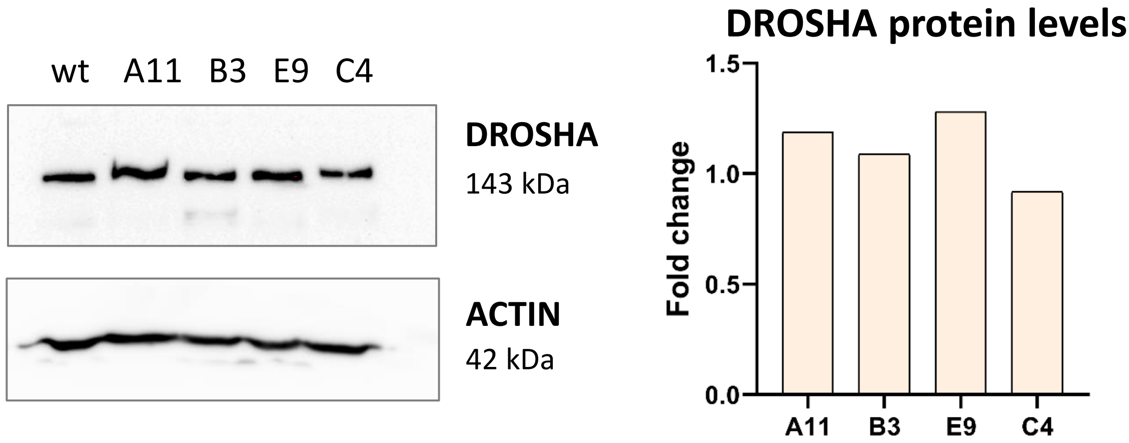

Supplementary Figure S7. Expression of DGCR8 and Drosha in the monoallelic mutant single cell clones. (**a**) Another biological replicate of relative mRNA expression levels; mean ± SD values of technical replicates are shown. (**b**) A biological replicate of DGCR8 protein level measurement in the clones relative to the parental HuES9 (WT) cell line. (**c**) A biological replicate of Drosha protein level measurement in the clones relative to the parental HuES9 (WT) cell line. For (**b**) and (**c**), Western blot images are on the left, quantification results are on the right.
