# Supplementary material for: Partial Disturbance of Microprocessor Function in Human Stem Cells Carrying a Heterozygous Mutation in the DGCR8 Gene
Source: Genes (Basel). 2022 Oct 23;13(11):1925. doi: 10.3390/genes13111925 (PMC9689658; doi:10.3390/genes13111925)
Supplement: Supplementary file 1 [file genes-13-01925-s001.zip › Table S1 Ree et al.pdf]

Primers for SYBR Green assay

Primer name	Sequence	Application
C19MC-1-For	5’- TAACTGGTGGTCAGCTTACAATGTG	C19MC-1 pri-miRNA quantification
C19MC-1-Rev	5’- CGAAAGTTGCATCTGTACGACTGG	C19MC-1 pri-miRNA quantification
C19MC-2-For	5’- TTCGCTCTGCTCCTGGCTGTC	C19MC-1 pri-miRNA quantification
C19MC-2-Rev	5’- GGCCTGGGAAGCTGCCTTCGGC	C19MC-2 pri-miRNA quantification
C19MC-3-For	5’- GGAATTAGAGGTGTGAGCCGC	C19MC-3 pri-miRNA quantification
C19MC-3-Rev	5’- CAACAGCAATGGATGGAGAGC	C19MC-3 pri-miRNA quantification
miR-512-For	5'- TGGCACTCAGCCTTGAGGGCACTT	Unprocessed pri-miRNA 1
miR-512-Rev	5'- TGGCGCAGAACAAGCACCACGG	Unprocessed pri-miRNA 1
miR-517c-For	5'- GCAAGAAGATCTCAGGCAGTGACCC	Unprocessed pri-miRNA 2
miR-517c-Rev	5'- GCCACCCACTCTAGTCTGGGCAA	Unprocessed pri-miRNA 2
miR-522-For	5'- GCAAGAAGATCTCAGGCTGTGTCCC	Unprocessed pri-miRNA 3
miR-522-Rev	5'- ACCGCACTCCAGTTTGGGCAGC	Unprocessed pri-miRNA 3
